# Management of Endodontic and Periodontal Lesions: the Role of Regenerative Dentistry and Biomaterials

**DOI:** 10.3390/dj8020032

**Published:** 2020-04-03

**Authors:** Marco Tatullo, Francesco Riccitiello, Sandro Rengo, Benedetta Marrelli, Rosa Valletta, Gianrico Spagnuolo

**Affiliations:** 1 Department of Medical Sciences, Neurosciences and Sense Organs, University of Bari, 70124 Bari, Italy; 2 Department of Neurosciences, Reproductive and Odontostomatological Sciences, University of Napoli Federico II, 80131 Naples, Italy; riccitie@unina.it (F.R.); rosa.valletta@unina.it (R.V.); gianrico.spagnuolo@gmail.com (G.S.); 3 Department of Health Sciences, Magna Graecia University of Catanzaro, 88100 Catanzaro, Italy; sanrengo@unina.it; 4 Marrelli Health-Healthcare Center, St. Enrico Fermi, 88900 Crotone, Italy; benedetta.marrelli@calabrodental.it

**Keywords:** regenerative dentistry, dental stem cells, biomaterials, dental materials, tissue engineering, translational medicine

## Abstract

Regenerative dentistry represents a novel interdisciplinary approach involving biomaterials, several molecules and mesenchymal stem cells (MSCs), preferably derived from oral tissues. The pivotal role of MSCs depends on the fact that they can differentiate into different cell lineages and have the strategic role to release bioactive substances that stimulate the renewal and regeneration of damaged tissues. The role of regenerative dentistry is promising in all the branches of dentistry: the most intriguing application is related to the management of endodontic and periodontal defects, overcoming the surgical approach and the implantology as a consequence of a poorly efficient therapeutic plan.

Regenerative dentistry represents a novel interdisciplinary approach, involving biomaterials, bioactive molecules, and mesenchymal stem cells (MSCs), preferably derived from oral tissues. Among these players, the pivotal role is carried out by MSCs; this depends on the fact that they can differentiate into different cell lineages and have the strategic commitment to release in the surrounding tissues several bioactive substances that stimulate the renewal and regeneration of damaged/injured tissues. On the other hand, novel biomaterials are biomimetic and easy to manage; smart materials can serve as a scaffold, where the efficient interaction between scaffold, cells and surgical bed should be able to induce and improve tissue healing and replacement. An ideal biomaterial should have good chemical stability, allowing the cell homing and MSC differentiation, avoiding cytotoxic conditions; finally, it should also be fully resorbable in a specific time period. 

## 1. The Guided Tissue Regeneration (GTR) in Periodontics 

Tissue engineering can be obtained with different techniques: the literature has reported interesting approaches over the past decades, and many of them are currently the gold standard in several clinical conditions. One of the most preferred techniques is the guided tissue regeneration (GTR). GTR consists in the use of biomaterials and scaffolds between the gingival tissue and the dental root; typically, this technique is able to stimulate the formation of new cement, increasing the quality of the soft tissue growth over the surgical site. In this context, GTR has been a pioneer, developing new conservative and regenerative techniques. Currently, several periodontal surgeons use semipermeable membranes on the dental area to be regenerated; this protocol promotes the formation of novel tissues surrounding teeth and improves the aesthetic quality of the zone to regenerate [1,2,3].

## 2. The Platelets Concentrates: PRP and PRF

The role of regenerative dentistry is promising in all the branches of dentistry: the most intriguing application is related to the management of endodontic and periodontal defects, overcoming the surgical approach and the implantology as a consequence of a poorly efficient therapeutic plan. 

At the end of the ‘70s, the biomedical companies have worked on the production of the first biomatrices derived from the human plasma, to make them easily producible and available on the market. Such matrices were called Platelet-Rich Plasma (PRP), and they were mainly made by a fibrin network able to mimic the last stages of the blood coagulation [4]. In more detail, PRP is characterized by a high concentration of autologous platelets obtained after blood centrifugation; PRP is rich in such growth factors involved in the healing of several injured tissues [5]. 

The platelet concentrates have been widely used and studied in oral and maxillofacial surgery. The most investigated surgical procedures were on patients requiring a maxillary sinus lift: the literature has clearly demonstrated that patients treated with PRP showed a significant improvement in bone healing, typically six months after the intervention, compared to control subjects treated without platelets concentrates [6]. PRP has also been used to support the bio-activation of dental implants inserted in the maxillary and mandibular regions, with somewhat promising effects [7].

A novel blood-derived product was also well reported in the literature over the last 20 years: it is called Platelet-Rich Fibrin (PRF) [8]. Similarly to PRP, PRF was developed and widely used in oral and maxillofacial surgery. A number of international studies on this matrix confirmed its important contribution to tissue healing, with a special impact on soft tissues. The matrix obtained by the PRF protocol is made from autologous fibrin, which has a tight network of fibers able to entrap leukocytes, platelets, and growth factors together. Such an enriched matrix can generate a faster and more accurate vascularization, as PRF release in the local environment molecules with angiotrophic, hemostatic and osteoconductive properties. The main proteins deputed to the new bone formation are released slowly and gradually over time. Thanks to its ability in the long-time release of growth factors, the PRF has replaced the previous protocol to obtain PRP. 

In dental fields, PRF is applied to a number of clinical and surgical cases. Interestingly, PRF can be used as a support in gingival regeneration or regrowth, or also in the guided bone regeneration involving peri-implant and periodontal bone defects [9]. The PRF matrix is characterized by some biological compounds able to accelerate the physiological wound healing; some of these compounds are highly important in bone tissue regeneration, such as the extracellular matrix-derived glycoproteins, and some growth factors (BMP2, BMP7, VGF). These last molecules have been found in the PRF-derived fibrin network up to 8–10 days after PRF engraftment, allowing their release over all these days [10]. Data reported in the literature have shown how the PRF membrane can induce and increase the long-term conservation of the crestal bone; more specifically, PRF has been demonstrated to play a central role in the healing of soft tissues around teeth and dental implants [11]. 

## 3. The Role of Growth Factors (GFs) and Mesenchymal Stem Cells (MSCs) in Oral Tissues Engineering

Growth factors (GFs) are molecules produced by various cell types and secreted in the surrounding tissues. GFs act as signaling elements that modulate and induce cells towards their development. 

GFs have been widely and differently used in oral and maxillofacial surgical protocols; among all the investigated molecules, the literature recognizes the main roles of platelet-derived growth factor (PDGF), base fibroblast growth factor (bFGF), insulin-like growth factor (IGF), vascular endothelial growth factor, growth factor-beta (TGFb) (VEGF) and bone morphogenetic proteins (BMPs) used in various cases of bone replacement and/or reconstruction. 

In this landscape, the most relevant GFs in bone tissue engineering are the bone morphogenetic proteins (BMPs), which are part of the large family of TGF-βs (growth and differentiation factors). The members of the BMP family are divided into at least four subgroups, according to their primary aminoacidic sequence: they promote the proliferation of mesenchymal stem cells and stimulate their chondrogenic and osteogenic differentiation; this is the main reason for their incredibe performance properties and their significant role in bone regeneration and repair. 

Mesenchymal stem cells (MSCs) have been largely used in the treatment of dental and bone tissue diseases during the last decades. The main role of MSCs has been focused on their ability to differentiate in specialized and adult cells able to support tissue repairing. The role of MSCs is closely related to the environment where they are cultured and/or grown: in fact, the role of the cell niche is in determining in the MSCs’ fate and behavior. Similarly, MSCs are typically conditioned by local molecules and GFs, reassessing the important and strategic role of this combination of MSCs–GFs in tissue regeneration. Furthermore, MSCs’ homing and engraftment are also key issues for dental and bone formation. Maxillary bone reconstruction has been widely improved thanks to the use of MSCs recruited from the bone marrow: these specific cells have been investigated on titanium scaffolds loaded with BMP-2; the MSCs–GFs–scaffold combination has produced smart and innovative scaffolds working on large defects, with critical-sized bone lacunas [12]. 

Similar studies were also carried out on adipose stem cells (ASCs): the seeding of ASCs onto a microvascular flap, in combination with beta-tricalcium phosphate (b-TCP) and BMP-2 showed promising results in the treatment of large and complicate jaw defects, up to 8 months after the graft implantation [13]. 

## 4. The Role of Biomaterials in Oral Tissues Engineering

Biomaterials for dental and periodontal applications can be obtained from different sources. Commonly, bone substitutes are obtained from animal bones, and the bovine bone is one of the most used and highest performing sources. Biomaterials are important for their ability to influence the quality, quantity and speed of the tissue regeneration process. MSCs are cells capable of differentiating towards different cell types; the differentiation of MSCs is influenced by the consistency of the surrounding extracellular matrix, which provides the cells with a mechanical signal. An interesting regenerative construct is based on dental pulp stem cells (DPSCs) seeded on hydrogel scaffolds. Hydrogels can be obtained from several sources: recently, a promising technique has obtained hydrogel scaffolds derived from decellularized and demineralized bovine bone (bECM). Several studies have been carried out, comparing hydrogels scaffolds obtained from bECM and collagen scaffolds. Most of the literature converges on the fact that MSCs seeded on bECM-derived scaffolds are mostly able to differentiate towards osteogenic cells. bECM-derived scaffolds are, in fact, able to stimulate a higher mineral deposition by an up-regulation of both osteogenic genes and markers related to bone formation, compared to what happens with collagen scaffolds [14,15]. An issue in regenerative and reparative medicine is represented by graft survival after implantation: the survival rate strongly depends on the quality of graft vascularization. Nevertheless, an adequate vascularization may ensure the adequate provision of growth factors and the proper homing of MSCs to the surgical graft, allowing the growth and development of the surrounding tissues. Recently, the scaffolds’ vascularization has been investigated by using a novel approach, called engineered cells without scaffolds (CSE): this technique works to create and provide an adequate environment for graft vascularization. In the CSE technique, the cells are organized in sheets and overlapped to improve the cell density; this is allowed by a better vascularization of the graft, able to ensure greater efficiency of the scaffold during its role to regenerate bone tissue [16].

## 5. Conclusions and Future Insights

Regenerative medicine aims to improve or repair the physiological function of tissues or organs through processes of replacement, engineering or regeneration. Dental treatments often require the replacement or repair of tissues that could be obtained by tissue engineering: we have discussed the main techniques to perform regenerative dentistry, involving stem cells, biomaterials and growth factors in combination or used alone [17,18,19,20]. Novel technologies and protocols have been demonstrated to be useful in bone and periodontal tissue regeneration. The management of endodontic and periodontal lesions recognizes the growing role of regenerative dentistry. The technique of tissue engineering has been simplified and improved overtime: this means that dental practitioners will be able, in the very near future, to perform tissue engineering in their own dental cabinets, avoiding failures due to infections or the operator’s bias [21,22,23,24]. The collection of stem cells can be obtained from several donor sites: the oral cavity hosts a number of sources of MSCs that are easily obtainable, and the harvested cells can undergo low manipulation to be seeded on innovative and easy-to-manipulate scaffolds based on polymers or hydrogels. MSCs seeded on the scaffolds proliferate and differentiate towards the tissue to be regenerated and replaced. One of the most important players to be carefully chosen is the type and material of the scaffold. Furthermore, modern scaffolds can be designed and customized on the basis of patients’ requirements. Regardless of their composition, modern scaffolds must ensure structural support to cells, provide a flexible environment and space for tissue remodeling, and they must also supply the growth factors. Scaffolds are typically biocompatible, usually porous, and they should also have appropriate mechanical strength and interconnectivity, to allow an efficient and suitable diffusion of fluids and substances. Innovative dental biomaterials are even more focusing on the presence of different layers with different structures and morphologies: recently, nanoporous and mesoporous silicon-based scaffolds have been used to culture DPSCs for periodontal repairing [25]. The good results obtained from biomimetic scaffolds and dental-derived MSCs allow researchers and clinicians to bet on such a combination for future therapies. 
